# Barriers and facilitators to HPV and meningococcal vaccination among men who have sex with men: a qualitative study

**DOI:** 10.1186/s12889-023-15847-w

**Published:** 2023-05-23

**Authors:** Justin Naidu, Andrea N. Polonijo

**Affiliations:** 1 Department of Sociology, University of California, Merced, 5200 North Lake Road, Merced, CA 95343 USA; 2 Department of Public Health, University of California, Merced, 5200 North Lake Road, Merced, CA 95343 USA; 3 Health Sciences Research Institute, University of California, Merced, 5200 North Lake Road, Merced, CA 95343 USA

**Keywords:** Community-based research, Human papillomavirus vaccination, Meningococcal vaccination, Men who have sex with men, United States

## Abstract

**Background:**

Men who have sex with men (MSM) have suboptimal uptake of human papillomavirus (HPV) and meningococcal vaccines. This study examines barriers and facilitators to HPV and meningococcal vaccination among MSM in a large, racially/ethnically diverse, and medically underserved U.S. region.

**Methods:**

In 2020, we conducted five focus groups with MSM living in the Inland Empire, California. Participants discussed (1) their knowledge about and attitudes toward HPV, meningococcal disease, and related vaccines; and (2) factors that would encourage or discourage vaccine uptake. Data were systematically analyzed to identify salient barriers and facilitators to vaccination.

**Results:**

Participants (*N* = 25) had a median age of 29. Most were Hispanic (68%), self-identified as gay (84%), and had college degrees (64%). Key barriers to vaccination included: (1) limited awareness and knowledge about HPV and meningococcal disease, (2) reliance on mainstream healthcare providers for vaccine information, (3) stigma and reluctance to disclose sexual orientation, (4) uncertainty about health insurance coverage and vaccine costs, and (5) distance and time required to access vaccines. Key facilitators to vaccination were: (1) vaccine confidence, (2) perceived severity of HPV and meningococcal disease, (3) bundling vaccination into routine healthcare, and (4) pharmacies as vaccination sites.

**Conclusions:**

Findings highlight opportunities for HPV and meningococcal vaccine promotion, including targeted education and awareness campaigns for MSM, LGBT inclusivity training for healthcare providers, and structural interventions to improve vaccine accessibility.

## Background

Gay, bisexual, and other men who have sex with men (MSM) experience elevated rates of vaccine-preventable human papillomavirus (HPV) and meningococcal disease [1–4]. HPV is a sexually transmitted infection that can cause anogenital warts and anogenital and oropharyngeal cancers [2]. The estimated prevalence of anal HPV infection among U.S. MSM is > 80%, while the incidence of HPV-related anal cancer among MSM is approximately 20 times greater than among heterosexual men [2, 5]. Meningococcal disease is a rare but serious bacterial infection spread mainly through saliva and close contact [6]. The disease has a sudden onset, can cause kidney and neurological system damage, hearing and limb loss, and other serious complications, and has a high case-fatality rate of 10–20% [6]. Several clusters of meningococcal disease due to serogroup C have been reported among MSM in the United States in recent years (e.g., in Los Angeles County, Chicago, New York City, Florida) [1, 3]. Though the prevalence and epidemiology of meningococcal disease among U.S. MSM are poorly documented [1], it is estimated that MSM have four times greater risk of contracting the disease compared to non-MSM [3]. The burden of both HPV and meningococcal disease is even greater among MSM living with HIV [3, 4].

Effective vaccines exist to protect against HPV and meningococcal disease but uptake among MSM is suboptimal [7]. The U.S. Advisory Committee on Immunization Practices (ACIP) recommends two or three doses of the 9-valent HPV vaccine for everyone by age 26 and the vaccine is approved for and available to adults up to age 45 [8]. ACIP also recommends persons of any age—including MSM—who are at elevated risk of meningococcal disease due to underlying health conditions such as HIV or proximity to an outbreak or infected person receive the quadrivalent meningococcal conjugate vaccine if they have either never been vaccinated or were vaccinated more than five years ago [9]. A 2021 systematic review and meta-analysis estimated MSM’s rates of immunization against both HPV and meningitis C to be around 40%, though uptake varies by several factors including age, location, socioeconomic status, race, and ethnicity [7].

Identifying barriers and facilitators to vaccination is an important step toward improving vaccine uptake. Previous research has demonstrated that healthcare provider recommendation and disclosure of sexual orientation to healthcare providers are important facilitators to HPV vaccination among MSM, while out-of-pocket costs, lack of knowledge, safety and efficacy concerns, sexual orientation-related stigma, inaccessibility, and inconvenience have been identified as important barriers to HPV vaccine uptake for this group [10–12]. Though research on meningococcal vaccine uptake among U.S. MSM is scarce, studies of subpopulations of MSM in Chicago and Los Angeles suggest costs, medical mistrust, lack of knowledge, and lack of perceived risk deter meningococcal vaccination, while vaccine confidence, perceived importance, and knowledge of others who have been vaccinated facilitate uptake [13–15].

Eliminating health disparities, such as those observed in HPV and meningococcal disease among MSM, requires tailoring research and interventions to be relevant to and effective in disadvantaged communities [16]. Our study aimed to identify regionally specific barriers and facilitators to HPV and meningococcal vaccination among MSM living in the Inland Empire, California. This 27,000mi^2^ Southern California region is larger than 10 U.S. states and has a population of > 4.5 million [17]. Inland Empire residents are racially/ethnically diverse (> 50% identify as Hispanic or Latinx and 17% identify as part of a non-Hispanic minoritized racial group) and are less likely to have completed college and more likely to experience poverty compared to California residents overall [17–19]. A severe shortage of healthcare providers exacerbates barriers to healthcare access for residents in this region [17].

## Methods

### Recruitment and data collection

The University of California, Riverside Socio-Behavioral Institutional Review Board approved the study, which was part of a larger community-based research project assessing MSM’s preferences for bundling rapid HIV testing with HPV and meningococcal vaccination [20]. In the fall of 2020, we conducted focus groups with English- and Spanish-speaking MSM aged 18 + who lived in the Inland Empire to elucidate (1) community-level perceptions about HPV and meningococcal vaccination (the focus of the current study) and (2) attitudes toward promoting these vaccines during rapid-HIV testing (findings published elsewhere) [20].

Focus groups were ideal for this study as they allowed us to explore community-level norms and prompt the emergence of ideas that may not have surfaced in an individual interview format [21]. Participants were recruited through LGBT social support groups, social media posts (Facebook, Instagram, Twitter), and listserv announcements distributed via community health and LGBT organizations. Participants verified their eligibility, registered for the study, provided consent, and completed a short demographic survey via Qualtrics. Thirty-two registrants met the study’s inclusion criteria and were invited to a focus group; 25 participated and received a $30 USD gift card as compensation.

We conducted five 90-min focus groups, three in English and two in Spanish, with approximately five participants in each group. All groups were conducted virtually, via Zoom. Participants used pseudonyms and were asked not to disclose personally identifying information. Trained moderators who identified as MSM led the focus groups using a semi-structured interview guide, which has been previously published [20]. Respondents were first asked a set of questions about HPV and HPV vaccination and then asked a separate set of questions about meningococcal disease and meningococcal vaccination. Relevant questions for the current analyses focused on (1) knowledge about HPV, meningococcal disease, and related vaccines (i.e., “What do you think of when you hear HPV/meningitis?”; “What have you heard about HPV/meningitis vaccines?”) and (2) attitudes toward HPV and meningococcal vaccines (i.e., “What do you think about being vaccinated against HPV/meningitis?”; “What reasons would you have for being/not being vaccinated against HPV/meningitis?”), and (3) factors that would encourage or discourage vaccine uptake (i.e., “What might make it easy/difficult for you to access HPV/meningitis vaccines?”). Focus group recordings were transcribed verbatim and Spanish transcripts were translated into English by a bilingual research assistant.

### Data analysis

We analyzed the focus group transcripts in Microsoft Excel using the rigorous and accelerated data reduction technique [22]. Two questions guided our analysis: (1) How do MSM’s knowledge and attitudes about HPV, meningococcal disease, and associated vaccines function as barriers and/or facilitators to vaccination? (2) What are MSM’s perceived barriers and facilitators to HPV and meningococcal vaccination? The first author open-coded the focus group transcripts, combined codes into categories, and identified emergent themes summarizing recurrent patterns in the data [23]. The second author then reviewed the complete focus group transcripts alongside the first author’s codes, themes, and representative quotes to verify interpretations. Any discrepancies in coding were resolved via discussion. No new themes emerged from the data upon analysis of the fifth focus group, suggesting we reached data saturation.

## Results

Key participant demographics are detailed in Table 1. Twenty-three participants were between 22 and 45 years old and thus eligible for both HPV and meningococcal vaccination; the remaining two participants were over age 45 and only eligible for meningococcal vaccination. Most participants (68%, *n* = 17) were Hispanic, identified as gay (84%; *n* = 21), had completed a college degree or higher level of education (64%, *n* = 16), and were not living with a known HIV diagnosis (76%, *n* = 19). Seventy-two percent (*n* = 18) of all participants reported never being vaccinated against meningococcal disease and 68% (*n* = 17) had never been recommended this vaccine by a healthcare provider. Seventy percent (*n* = 16) of the 23 participants who were eligible for HPV vaccination reported never receiving this vaccine and 57% (*n* = 13) had never been recommended this vaccine by a healthcare provider.

**Table 1:** Demographic characteristics of focus group participants (*N* = 25)

	*n* or Median	Percent or Range
Age	29	22 – 57
Household income in 2019	45,000	0 – 120,000
*Ethnicity*		
Asian/Pacific Islander	3	12%
Hispanic	17	68%
White (non-Hispanic)	2	8%
Other or multiple races/ethnicities	3	12%
*Sexual Orientation*		
Bisexual	4	16%
Gay	21	84%
*Education*		
< College degree	9	36%
≥ College degree	16	64%
Has health insurance	22	88%
Living with HIV	6	24%

All participants self-identified as “men who have sex with men”. To protect participant confidentiality, we do not report transgender status or more refined racial/ethnic categories

### Barriers to vaccination

Qualitative analyses revealed that barriers to HPV and meningococcal vaccination closely overlapped. These included: (1) limited awareness and knowledge, (2) reliance on mainstream healthcare providers for vaccine information, (3) stigma and reluctance to disclose sexual orientation, (4) uncertainty about health insurance coverage and vaccine costs, and (5) distance and time required to access vaccines. We detail these barriers below and provide exemplary supporting data in Table 2.

**Table 2:** Key barriers to HPV and meningococcal vaccination and exemplary quotes

Barriers	Exemplary Quotes
*Limited awareness and knowledge*﻿	
…about HPV and HPV vaccination	“*All I know is it’s [HPV is] a thing that you can get. Like it’s not high on my radar of STDs [sexually transmitted diseases] or anything else.*” (John, 40s, White, ≥ college degree) *“I have not had relationships with someone with a uterus and from what I understand, it [HPV] is more of a risk when you have relationships with a person with a uterus.”* (Quin, 20s, Hispanic, ≥ college degree)
…about meningococcal disease and meningococcal vaccination	*“Meningitis…when I first heard about it, I was clueless, until I looked it up and saw what it was. … it is the… the one, disease, we’ll use that word, that you do get as a college freshman if you choose to live in the dormitories. It is very common amongst college freshmen.”* (Ava, 20s, multi-racial, ≥ college degree) *“In all honesty, I know almost nothing about meningitis.”* (Quin, 20s, Hispanic, ≥ college degree)
Reliance on mainstream healthcare providers	*“When I was in San Diego, I had a gay doctor because I was in a good community and now I’m out here in the wilderness in the barbaric lands of the Inland Empire and I wouldn’t even know where to find a gay doctor anymore.”* (John, 40s, White, ≥ college degree) *“I would like to know from my doctor, how effective the (meningococcal) vaccine is for people in my age group... it would be good for me to have that information.”* (Alex, 50s, Asian/Pacific Islander, ≥ college degree)
Stigma and reluctance to disclose sexual orientation	*“[I told my doctor] I’m a gay man so if there’s any special risk factors from anything that you can let me know. It freaked him out, he left, he was never available again to meet with me.”* (John, 40s, White, college degree) *“I think it is the stigma, I think in the Latino community we do not talk much about issues especially sexual health. So it is, in my family we never talk about anything sexual. All of the sex ed happens at school or on the internet or by friends”* (Boots, 20s, Hispanic, ≥ college degree)
Uncertainty about health insurance coverage and vaccine costs	*“…the other day I went to be tested for HIV here in San Bernardino and I saw that a person—they didn't want to give her a blood test because she didn't have insurance. Well, I had insurance, but the insurance company didn't want to pay, so that's what comes to mind is access to this [meningococcal] vaccine.”* (Xochipili*,* 20s, Hispanic, ≥ college degree) *“I know people who, they wanted to receive it [HPV vaccine], but the insurance did not cover it. I think they told me it would cost them like six hundred dollars because that's three and each dose was worth about one hundred and eighty or something like that.”* (Boots, 30s, Hispanic, ≥ college degree)
Distance and time required to access vaccines	*“The clinic that was telling me to get vaccinated did not offer the HPV vaccine. So they were telling me to go to this other place that, where I could get it…even though I would want to get the HPV vaccine, at the time, I just wasn’t able to… I had no reliable means of transportation.”* (Wario, 20s, Hispanic, ≥ college degree) *“I have a very demanding job that makes seeing medical professionals difficult.”* (Ailesq, 20s, Hispanic, ≥ college degree)

#### Limited awareness and knowledge

##### ***About HPV and HPV vaccination***

Participants were generally aware that anyone who was sexually active could carry the HPV virus and that HPV infection could lead to anogenital warts in all genders and cancers among cisgender women. However, very few participants were aware that men can develop HPV-related cancers or that MSM have an elevated risk of HPV-related anal cancer. As Miguel (20s, Hispanic, < college degree) asserted,* “With women, cancer is more severe, there are more cases of cancer caused by the [HPV] virus. And for men, there are certain types of the HPV virus that do not cause cancer.”* The assumption that HPV-related cancers only affect cisgender women contributed to a general lack of concern around HPV compared to other sexually transmitted infections, as Boots (30s, Hispanic, ≥ college degree) summarized:

“*There is a lot of attention about sexually transmitted diseases that are more common: gonorrhea, chlamydia, syphilis… I don’t know if this [HPV] is actually one of the diseases that is thought of in the same way in our world.”*

Though most participants were aware that all genders were eligible for HPV vaccination, several falsely assumed they were “too old” to be vaccinated. As highlighted by Ailesq (20s, Hispanic, ≥ college degree), who was currently eligible for HPV vaccination: *“I was interested but had aged out of the vaccine efficacy range by the time I even learned a vaccine existed.”*

##### ***About meningococcal disease and meningococcal vaccination***

Participants who had been recommended meningococcal vaccines to attend college were generally aware that meningococcal disease is transmitted through close contact and can cause inflammation of the brain and spinal cord. However, many other participants stated they knew nothing about meningococcal disease or how it is transmitted. For example, Wario (20s, Hispanic, ≥ college degree) asked, *“What is meningitis?… when I think of it, it reminds me of something that was eradicated like back in the days of polio.”* Participants had differing perceptions of which subpopulations were at elevated risk of meningococcal disease (e.g., “college students”, “older people”) and only one participant acknowledged any risk specific to MSM. As Chuy (50s, Hispanic, < college degree) shared: *“I believe that like all human beings, we are all exposed [to meningococcal disease], everyone without exception, they can be gay, they can be heterosexual. They can be young or old, boy or girl.”* Multiple participants did not view themselves as candidates for meningococcal vaccination because they assumed they had been immunized in childhood and were unaware of current ACIP booster guidelines. As Roy (20s, Hispanic, ≥ college degree) explained:

“*I think I got this vaccine when I was like a little boy, when I was a child, cuz I really didn’t think much of it after. It was like, ‘Oh I already have it,’ then, that’s off the checklist when I go to college.”*

#### Reliance on mainstream healthcare providers for vaccine information

Participants reported a dearth of healthcare resources for MSM in the Inland Empire. Consequently, many recounted relying on mainstream healthcare providers—rather than providers designated as LGBT-friendly—for vaccine information. Though several participants reported wanting to hear about recommended vaccines and associated safety and effectiveness from healthcare providers, most had never discussed HPV or meningococcal vaccination with them. Ailesq (20s, Hispanic, ≥ college degree) shared:

*“I think medical professionals need to be more on the ball about suggesting these things [vaccines]. I do not even remember being offered the meningitis vaccine… It seems weird to basically get radio silence from my treatment providers on these important vaccines.”*

In the absence of conversations about HPV and meningococcal vaccination with healthcare providers, some participants reported assuming they were not candidates for either vaccine. As John (40s, White, ≥ college degree) shared, *“I’m a reasonable citizen. I go to my doctor and I say ‘Is there anything I should know?’ and if my doctor doesn’t say to get anything, I won’t get it.”*

#### Stigma and reluctance to disclose sexual orientation

Several participants reported previous experiences of stigmatization in healthcare settings due to their sexual orientation and/or HIV status. As a result, some were reluctant to seek vaccines in clinical settings. As Chuy (50s, Hispanic, < college degree) stated:

*“… stigma has a lot to do with this ... The reason is that, if I go and enter a clinic to get a vaccine, maybe everyone knows that I am [HIV] positive, or they will point me out. And that is a factor that can affect [vaccine uptake] of course.”*

Other participants reported reluctance to disclose their sexual orientation to healthcare providers, which they felt interfered with their ability to engage in shared clinical decision-making conversations about relevant vaccinations. For example, Atlus (20s, Hispanic, < college degree) shared:

*“a lot of people just aren’t really comfortable talking about their sexuality or really just opening up. Especially to their physicians… it kind of feels like they’re not as open or really accommodating to your needs specifically as a gay man…a lot of the questions that you wish you could ask [healthcare providers] comfortably about these sorts of things you just kind of keep to yourself.*”

#### Uncertainty about health insurance coverage and vaccine costs

Although 88% of participants had health insurance, many were unsure of whether their insurance covered HPV or meningococcal vaccination. This uncertainty, coupled with high anticipated out-of-pocket costs to obtaining vaccines, were noted as barriers to vaccination for several participants. Anticipation of high out-of-pocket costs were most pronounced for obtaining the HPV vaccine series. As Wario (20s, Hispanic, ≥ college degree) recounted:

*“I was told by a healthcare provider to go get vaccinated [against HPV]... they kept just telling me about how expensive it is… that was a real big turn off… it was just like ‘ah well, I know it’s important, but, I can’t afford this right now from how they’re framing it to me’ so, I just never really looked into it, even though I now have health insurance.”*

#### Distance and time required to access vaccines

Many participants noted anticipating the need to travel significant distances to obtain HPV or meningococcal vaccines and perceived this as a barrier to uptake for themselves and others in the community. Lack of access to either a private vehicle or reliable public transportation and limited flexible time away from work to commute to and attend vaccine appointments exacerbated this perceived barrier for some participants. As Boots (30s, Hispanic, ≥ college degree) emphasized.

*“If you do not live near the doctor or do not live near the place where the vaccine is administered, if you have to request the day off work, if you need to get transportation. Those can be obstacles [to vaccination].”*

### Facilitators to vaccination

Facilitators to vaccination were also similar across HPV and meningococcal vaccines and included: (1) perceived disease severity, (2) vaccine confidence, (3) bundling vaccination into routine healthcare, and (4) pharmacies as vaccination sites. We discuss these facilitators below and provide exemplary supporting data in Table 3.

**Table 3:** Key facilitators to HPV and meningococcal vaccination and exemplary quotes

**Facilitators**	Exemplary Quotes
*Perceived severity*	
…of HPV	*“HPV can cause cancer in men. It depends a lot on the immune system, it makes me think of talk at my university by representatives of the AIDS Healthcare Foundation… on the vulnerability to HPV-related cancer in men who are living with HIV and have a detectable viral load.”* (Miguel, 20s, Hispanic, < college degree) *“I want to be protected from getting HPV and having whatever it turns into. Like, if it develops into cancer, I’m concerned about that.”* (Wario, 20s, Hispanix, ≥ college degree)
…of meningitis	*“I know my freshman year of college, it did spark enough of a fear in me to opt out of living on campus and in a dormitory because I was like, ‘Oh my gosh! What if someone, what if my roommate, you know, can give me this disease?’… I remember my fear was actually very serious”* (Ava, 20s, multi-racial, ≥ college degree) *“I actually have had a friend who got it [meningococcal disease]… I actually know it can be quite severe. To the point where I’ve seen actually, it can be deadly even. It can cause, like, whole body, like, sepsis.”* (Kaviyon, 20s, Asian/Pacific Islander, ≥ college degree)
Vaccine confidence	*“So if there was a vaccine and I needed it, I would go get it. That’s the end of the story.”* (John, 40s, White, college degree) *“I think it's [HPV vaccination] a good thing also. I mean, if it's going to prevent, you know, genital warts, cancer, like why not just get vaccinated?”* (Charlie, 20s, Hispanic, ≥ college degree)
Bundling vaccination into routine healthcare	*“Typically when I go get a vaccine for something, I’ll ask if there’s anything else I would get while I’m here. Sometimes I feel like I’ll get this list of a couple things like three vaccinations, I’ll go in and I’ll take those three.”* (Atlus, 20s, Hispanic, < college degree) *“Bundling things together is always efficient. Especially when there are barriers to [healthcare] access cuz you don’t want a patient to come back three, four times, versus, it’s more likely if they’re there in that first visit and you can capture all the care that you need and be that point of care initially.”* (Kaviyon, 20s, Asian/Pacific Islander, ≥ college degree)
Pharmacies as vaccination sites	*“I know that there are doctors who do not have the capacity to give these vaccines to their clients. And I know that sometimes they refer people to pharmacies. There are pharmacies that do offer them. And if the insurance covers it, they can get it at like CVS [pharmacy].”* (Boots, 30s, Hispanic, ≥ college degree) *“I got my vaccination at Walmart… behind that fold thing…. the customers around you don’t know what vaccine you’re getting. They could probably think you’re getting the flu vaccine… I have privacy there… even though it’s very public and open. The staff there is nice.”* (Roy, 20s, Hispanic, < college degree)

#### Perceived severity

##### ***Of HPV***

Despite limited baseline awareness and knowledge about HPV, through the course of the focus group discussions, participants generally concluded that HPV could have severe cancer-related consequences for MSM and particularly MSM living with HIV. Some participants specifically cited wanting to prevent severe cancer-related outcomes as the primary reason why they had previously sought or would plan to seek HPV vaccination in the future. For example, Benjamin (30s, other race/ethnicity, < college degree) recounted how knowing someone who had died of HPV-related cancer motivated him to seek out HPV vaccination for himself:

*“I got my HPV shot when I was 26…because my sister’s mother had died of HPV cancer. She caught it [HPV] from someone else when she was having sex… So I know that, you know that can happen but, I mean it was a good thing I got mine [HPV vaccination] done when I started being sexually active.”*

##### ***Of meningococcal disease***

Participants who were knowledgeable about meningococcal disease emphasized the severe complications associated with the disease, such as sepsis, brain swelling, and death. These complications, combined with the highly contagious nature and often sudden onset of meningococcal infection, were cited as sources of fear for multiple participants. For example, Chuy (50s, Hispanic, < college degree) shared:

*“…the strange thing is that this meningitis develops so aggressively that I can be fine, in a matter of hours I can present symptoms of exaggerated headache or bad vision… And the person can, depending on their immune system, can lose their life in a matter of two, three hours. So that's the dangerous thing, that's why I talk about how I'm very scared.”*

The possibility of experiencing severe complications was framed as justification for becoming vaccinated, even among participants who had low knowledge and awareness about meningococcal vaccination prior to joining the focus group. As Wario (20s, Hispanic, ≥ college degree) emphasized:

*“I feel… pretty firmly supportive of it [meningococcal vaccination] … having heard [meningococcal disease] can lead to things like brain swelling. That doesn’t really sound very fun, and death. I don’t want to die…. I’d want to be vaccinated if I haven’t already.”*

#### Vaccine confidence

Though vaccine hesitancy was expressed by two participants, the vast majority reported positive attitudes toward vaccination, agreed that HPV and meningococcal vaccines would be effective against severe disease outcomes, stated they did not have any specific safety concerns, and reported being open to being vaccinated. As Atlus (20s, Hispanic, < college degree) shared, *“Honestly any sort of vaccination that would prevent you from getting diseases in the future is a good reason to get them … I see it [vaccinations] as more of a beneficial thing.”*

#### Bundling vaccination into routine healthcare

Multiple participants noted that they would be more apt to be vaccinated against HPV or meningococcal disease if these vaccinations were integrated into routine healthcare encounters or bundled with appointments for other vaccines. For example, when asked what might help facilitate access to HPV or meningococcal vaccines for MSM in the community, John (40s, White, ≥ college degree) stated:

*“If I was given my flu vaccine, that would be a great time to tell me: ‘Hey, there’s some other things you might want to check out’….At my annual physical, that would be a great time to say: ‘Hey, this year you need a colonoscopy and you need to consider these four vaccines’… Tell me what the heck needs to be done, I’ll get it done.”*

This bundled approach was noted as being particularly important for facilitating vaccine uptake given the scarcity of healthcare resources in the Inland Empire region and the associated distance and time-related barriers to accessing vaccines.

#### Pharmacies as vaccination sites

Multiple participants reported positive experiences accessing flu vaccinations in pharmacies and stated this would be a preferred place to receive HPV or meningococcal vaccines. Compared to medical offices, pharmacies were often perceived to be closer in proximity, offer a wider range of hours for scheduling vaccine appointments, and have lower associated out-of-pocket costs. As Kaviyon (20s, Asian/Pacific Islander, ≥ college degree) emphasized:

*“I personally think a Walgreens or CVS [pharmacy] would be much more accessible than a doctor’s office… I tried scheduling an appointment [with my doctor] recently and the first available one is in like three months… and then you have co-pays associated with going to the doctor’s office versus if you could just walk in to like a CVS [pharmacy] and get the shot.”*

## Discussion

MSM’s vulnerability to HPV and meningococcal infection may be compounded when they face a scarcity of healthcare resources, such as in California’s Inland Empire [20]. This study aids in understanding the barriers and facilitators to HPV and meningococcal vaccination among MSM in the region, which may also be applicable elsewhere. Our findings point to three key recommendations for promoting HPV and meningococcal vaccine uptake among MSM.

First, limited awareness and knowledge about HPV, meningococcal disease, and related vaccines was the most prominent barrier to vaccination among MSM in our study. Low levels of knowledge and awareness surrounding susceptibility to and consequences of these infections have been identified as barriers to vaccination in studies of MSM in other regions [15, 23–26]. Multiple theories of health behavior change suggest perceived issue salience and susceptibility motivate health behavior adoption [27]. Hence, tailored and targeted public education and awareness campaigns highlighting MSM’s elevated risk of HPV and meningococcal disease, the potential for severe disease outcomes, and ACIP vaccine recommendations, may be one way to bridge vaccine knowledge gaps, increase awareness of vaccine recommendations, and drive vaccine uptake.

Second, echoing previous research [7, 15], MSM in our study looked primarily to healthcare providers to initiate vaccine recommendations. Though disclosure of sexual orientation to healthcare providers is a known facilitator to vaccination among MSM [7, 11, 12, 28, 29], limited access to designated LGBT-friendly healthcare providers in the region and fears of stigmatization deterred many MSM in our study from disclosure. The provision of LGBT inclusivity training for healthcare providers may help to encourage such disclosure, given this study and others [30, 31] have identified a lack of LGBT cultural competency in the Inland Empire region. Healthcare provider education focused on vaccine recommendations for MSM will likely also be important for encouraging providers to discuss HPV and meningococcal vaccines with their eligible patients [32, 33].

Third, our findings complement previous studies that highlight a variety of structural obstacles to HPV and meningococcal vaccination [14, 26, 29]. The Affordable Care Act mandates that health insurance plans include coverage for routine HPV and meningococcal immunizations without cost-sharing [34], yet uncertainty about health insurance coverage and high anticipated out-of-pocket costs were key barriers to uptake. Clear messaging surrounding vaccine-related health insurance coverage and initiatives that make HPV and meningococcal vaccines available to all eligible MSM free-of-charge may help address perceived cost-related barriers. Lack of flexible time to schedule vaccine appointments and distance from healthcare providers were also significant barriers to vaccination in our study. California law authorizes pharmacists to administer immunizations [35] and pharmacies were a preferred vaccination site for many MSM in our study. Hence, pharmacies could be leveraged for targeted HPV and meningococcal vaccine promotion and help to mitigate time, distance, and transportation-related barriers to uptake. As suggested by our participants, integrating HPV and meningococcal vaccination into routine healthcare encounters, such as annual physicals or flu vaccine appointments, may also mitigate barriers to vaccine access by eliminating the need for repeated office visits.

### Limitations

This study has several limitations. Data were acquired through focus group discussions and some participants tended to contribute more to conversations than others. Some participants may have been hesitant to disclose discordant opinions in a group setting, resulting in responses that were not entirely honest. While numerous previous studies have successfully employed focus groups to investigate health-related topics among diverse samples of MSM [14, 24, 25, 36], we acknowledge that sexual orientation-related stigma likely deterred some eligible MSM from choosing to participate in this study and thus the findings do not represent the perspectives of these individuals. Though we recruited a racially/ethnically diverse sample of MSM and conducted focus groups in English and Spanish, the study was limited to 25 participants and findings cannot be generalized to all MSM in the Inland Empire or beyond. Finally, the majority of our participants were college educated, and we might expect more pronounced barriers to vaccination to have emerged from a less educated sample.

## Conclusions

MSM remain under-vaccinated against HPV and meningococcal disease. Our study reveals that MSM in California’s Inland Empire face several barriers to HPV and meningococcal vaccination, ranging from limited awareness and knowledge about these diseases and related vaccine recommendations, to stigma surrounding sexual orientation disclosure, to structural barriers to vaccine access. However, this group holds generally positive evaluations of vaccination. Educational interventions emphasizing HPV and meningococcal disease susceptibility among MSM and ACIP vaccine recommendations may help alleviate awareness and knowledge gaps. The provision of LGBT inclusivity training for healthcare providers may further aid in encouraging sexual orientation disclosure and vaccine promotion for MSM in clinical settings. Finally, interventions designed to offset the perceived costs of vaccination, bundle vaccination into other healthcare encounters, and promote vaccination via pharmacies may help alleviate structural barriers to access.

## Data Availability

Supporting data is not publicly available due to ethical restrictions in place to protect the privacy of research participants, who did not provide consent for their data to be shared publicly. Inquiries related to supporting data availability should be directed to the principal investigator of the study (ANP) at apolonijo@ucmerced.edu.

## Abbreviations

ACIP: Advisory Committee on Immunization Practices
HPV: Human papillomavirus
HIV: Human immunodeficiency virus
LGBT: Lesbian, gay, bisexual, and transgender
MSM: Men who have sex with men
US: United States
